# Preserved Self-Evaluation in Amnesia Supports Access to the Self through Introspective Computation

**DOI:** 10.3389/fnhum.2016.00462

**Published:** 2016-09-16

**Authors:** Aurelija Juskenaite, Peggy Quinette, Mickaël Laisney, Marie-Loup Eustache, Béatrice Desgranges, Fausto Viader, Francis Eustache

**Affiliations:** ^1^Institut National de la Santé et de la Recherche Médicale (INSERM), U1077Caen, France; ^2^UMR-S1077, Université de Caen-NormandieCaen, France; ^3^UMR-S1077, École Pratique des Hautes Études (EPHE)Caen, France; ^4^ U1077, Centre Hospitalier Universitaire de CaenCaen, France; ^5^Service de Neurologie, Centre Hospitalier Universitaire de CaenCaen, France

**Keywords:** amnesia, episodic memory, trait self-knowledge, self-evaluation, self-representations, identity

## Abstract

Encounters with new people result in the extraction and storage in memory of both their external features, allowing us to recognize them later, and their internal traits, allowing us to better control our current interactions with them and anticipate our future ones. Just as we extract, encode, store, retrieve and update the representations of others so, too, do we process representations of ourselves. These representations, which rely on declarative memory, may be altered or cease to be accessible in amnesia. Nonetheless, studies of amnesic patients have yielded the surprising observation that memory impairments alone do not prevent patients from making accurate trait self-judgments. In this review article, we discuss prevailing explanations for preserved self-evaluation in amnesia and propose an alternative one, based on the concept of introspective computation. We also consider molecular and anatomical aspects of brain functioning that potentially support introspective computation.

In this review article, we discuss the literature on the representations people have of themselves, focusing on representations of self-personality traits. The latter are usually assessed using self-evaluation tasks, where participants are provided with a list of traits and asked to rate their self-descriptiveness. Self-evaluation on a set of traits involves retrieving personal information from semantic as well as episodic memory. Surprisingly, studies have shown that patients with semantic and/or episodic memory impairments are still able to provide accurate trait self-judgments. In this article, we review these clinical cases, as well as possible explanations for preserved self-evaluation in amnesia provided in the literature. In a different section, we describe an alternative cognitive operation that we called *introspective computation* that may be used to judge the self-descriptiveness of personality traits. This mental operation, which does not rely on personal information stored in long-term memory, may allow patients to provide accurate judgments of their personality traits despite their amnesia. We also discuss cognitive operations, including introspective computation, used in self-evaluation tasks, and their possible impairments in patients unaware of their personality changes.

## Trait Inference

To identify particular human beings, we have to rely on information regarding their external and/or internal attributes that we have already memorized. Contrary to the external attributes that are directly perceived through our senses, internal ones have to be inferred from observed behavior. Our ability to achieve this, known as *theory of mind* (Premack and Woodruff, 1978), allows us to appreciate people’s current moods or goals, thereby helping us adjust our present interactions with them. It also allows us to estimate their internal traits, which shed light on the causes of their current actions and their general behavioral tendencies, thereby helping us anticipate our future interactions with them (McCarthy and Skowronski, 2011).

Empirical evidence suggests that while trait inference can be consciously initiated by individuals, it nevertheless meets the criteria for an automatic process (McCarthy and Skowronski, 2011), as it usually takes place without any explicit intention, awareness or control (Winter et al., 1985). It occurs during the initial stages of behavioral observation (Winter and Uleman, 1984; Uleman et al., 1996) and may result in the inference of multiple and sometimes even competing traits (Ham and Vonk, 2011). Different social situations linking us to the individuals we observe may influence our tendency to infer traits. For example, in line with our general propensity to represent distant objects on an abstract level (Trope and Liberman, 2010), our tendency to infer traits increases with the psychological distance we perceive between ourselves and others, whether they are distant in space or time (Rim et al., 2009). This raises the question of whether we construct representations of our own traits, and if so, why.

## Positive Self-Perception

Just as we form and update representations of other people, so we form and update ones of ourselves, to help us regulate our interactions with others and take decisions about our personal lives. We construct self-representations via interactions with our environment (mostly other people), which communicates attributes to us through both direct (Bollich et al., 2011) and indirect feedback (Harris and Rosenthal, 1985). During the creation and updating of our self-representations, we can engage in self-assessment to increase the accuracy of our self-perceptions, and self-verification to confirm them. However, these motivations seem to be weaker than the motivation to possess a positive self-view, as people choose to process—and tend to remember and acknowledge—the positive information about themselves rather than the negative information, even when it is important for the self (for a review see Sedikides, 2007). A positive self-view can be achieved either by maintaining and elevating our positive features or by protecting them against threatening information.

If we are mentally healthy, we tend to see ourselves in a more positive light than we see others on a wide variety of desirable attributes. Needless to say, our belief of being above average (Williams and Gilovich, 2008), termed the *better-than-average* effect (Alicke, 1985; Brown, 1986), is not shared by others, who usually perceive us in a less positive light. Despite not being objective, our overly positive self-view has several advantages, the most important of which seem to be the attainment of relevant goals, and both psychological and physical wellbeing (for a review see Sedikides and Alicke, 2012).

## Assessment of Self-Representations

Studies designed to assess people’s explicit self-representations essentially use two kinds of tasks, involving either self-description or self-evaluation. In self-description tasks, individuals freely describe themselves, whereas in self-evaluation tasks, they are asked to assess themselves on a series of statements. The best-known *self-description* task is the Twenty Statements Test (Kuhn and McPartland, 1954), in which individuals give 20 different answers to the open-ended question “Who am I?” The precise content of these responses can vary considerably across individuals, but participants usually start by providing the social categories to which they belong, subsequently reporting what characterizes them as individuals, which for the most part includes their physical attributes, internal traits, and preferences. Analysis and interpretation of these open answers is, however, complex, for in addition to a high level of inconsistency across the scoring methods proposed in the literature, we still do not know how these self-descriptions are selected by individuals and which cognitive operations are at work. While there is some evidence to suggest that self-descriptions are essentially driven by a semantic type of self-knowledge (Grilli and Verfaellie, 2015), neither their importance (Trafimow and Finlay, 2001) nor their descriptiveness (Carpenter and Meade-Pruitt, 2008) can entirely account for their generation. Other hypotheses, such as chronic accessibility or contextual salience, still need to be tested.

The second type of task used to assess participants’ self-representations involves *self-evaluation* on a list of traits. In this type of task, participants are asked to rate each of the traits for self-descriptiveness on a Likert-type scale. The ratings are usually processed in such a way as to indicate the positivity of the self-judgments. Two functionally independent declarative memory systems can be separately or jointly activated during self-evaluation. Episodic memory is used to recall instances of specific behaviors exemplifying particular traits, while semantic memory provides direct access to traits represented in the form of general knowledge. The results of experimental studies using a task facilitation paradigm to infer the type of cognitive operation involved, on the basis of participants’ reaction times, suggest that in situations requiring self-evaluation on traits (e.g., *autonomous*), individuals initially access trait-consistent information, in the form of semantic self-knowledge (e.g., “I am an autonomous person”), as this allows for speedy decision making. If they do not possess trait-consistent semantic information, access to trait-inconsistent semantic information (e.g., “I am a dependent person”) may be sufficient to conclude that the trait under consideration does not apply to them (Klein et al., 1992). However, when information from a self-knowledge database is retrieved (e.g., “I am an autonomous person”), it usually triggers the retrieval of contrasting episodic memories (e.g., “When I bought my first video camera, I asked a friend to show me how to use it”), thus helping to limit its scope. It is worth noting that episodic memories matching the accessed trait self-knowledge are not retrieved (e.g., “The last time I encountered a problem with my video camera, I resolved it by myself”), probably to avoid information redundancy (Klein et al., 2001).

## Self-Evaluation in Amnesic Syndromes

When it comes to understanding the mental strategies that allow for accurate self-judgment, cases of patients with episodic or semantic memory impairments or with personality changes are particularly informative. Most of the studies examining these cases have used self-evaluation tasks, as these allow for greater standardization, and the cognitive mechanisms behind them are understood better than those behind self-description tasks. In the next section, we review studies reporting surprising observations of accurate self-judgment in patients with severe declarative memory impairments. We discuss the conclusions drawn by the authors of these studies and, where appropriate, suggest alternative interpretations.

Investigators of self-evaluation in the field of neuropsychology have primarily focused their attention on issues related to episodic memory. Several studies examining patients with episodic memory impairments have come to the surprising conclusion that the latter have no real impact on patients’ self-evaluation. One such patient, WJ, rendered temporarily amnesic by a head injury, was asked on two occasions to complete a self-evaluation task (Klein et al., 1996). She underwent her first testing session 5 days after the injury, at which point she was unable to recall episodic memories from the previous 6 months, corresponding to her first semester at university, although some memories that were older than 12 months could be recalled. WJ’s personal semantic knowledge, including that which she had acquired during her previous 6 months at university, was intact. The second testing session took place 3 weeks later, after she had recovered from her episodic memory impairments. Her performance on self-evaluation tasks while amnesic (first testing session) showed only a weak correlation between the university and high-school periods, indicating that her self-judgment in the present was not based on memories of her past. Moreover, her self-evaluation for the university period, performed while amnesic (first testing session), was significantly correlated with the one she performed after recovering from amnesia (second testing session), indicating that despite the loss of episodic memories, WJ was able to describe what she was like.

Even more convincing evidence of accurate self-judgment despite an inability to access episodic memories was obtained from Patient KC, who became permanently amnesic after a motorcycle accident (Tulving, 1993). In addition to a total loss of episodic memory, covering his entire lifespan, KC’s accident caused a profound change in his personality. More than 10 years after his head injury, he was asked to rate himself on a list of trait-adjectives. KC’s self-evaluation ratings were comparable to his mother’s ratings of his present—but not his past—personality, suggesting that KC’s self-judgment matched his current personality, and was not based on his previous knowledge about himself. As KC was incapable of recollecting any events that had been personally experienced since the accident, his ability to accurately report his own traits could not be supported by his episodic memory. Tulving therefore concluded that KC’s accurate self-evaluation was based on his updated “self-knowledge, belonging to the same category as all his other knowledge about the world” (Tulving, 1993, p. 155).

Following this reasoning, impairment in semantic memory (i.e., general knowledge about the world and the self) should compromise accurate self-evaluation. However, a study conducted among patients with semantic dementia nonetheless showed that these patients were able to complete the self-evaluation task, and their self-ratings were comparable to those of the control group (Duval et al., 2012). However, as the patients’ traits had not been rated by their relatives, the authors could not definitely conclude that patients with semantic dementia can accurately judge themselves.

This issue was investigated by Klein et al. (2002), who examined Patient DB, rendered permanently amnesic by anoxia following cardiac arrest. DB’s severe retrograde and anterograde amnesia affected his episodic memory, manifesting itself in his inability to recall a single specific event he had experienced either before or after the cardiac arrest. Moreover, while DB’s general level of intelligence seemed to be preserved, his knowledge of public as well as personal facts and events was somewhat impaired, albeit less severely than his episodic memory. Surprisingly, DB’s self-evaluation was accurate, as his self-ratings correlated significantly with his daughter’s ratings of him. By contrast, DB’s knowledge about his daughter’s traits seemed to have been affected by his illness, as the correlation between his ratings of his daughter and her self-ratings was nonsignificant. Taken together, it seems that while DB’s episodic and semantic memory impairments affected his knowledge of personal facts and other people’s traits, they nonetheless spared his ability to accurately rate himself. Klein et al. (2002) interpreted DB’s accurate self-evaluation despite episodic and semantic memory impairments as evidencing a dissociation between general semantic knowledge and semantic knowledge of one’s own traits. The latter, unlike knowledge about other people’s traits, appears to be extremely resistant to brain damage.

DB’s performance can be regarded in two ways, suggesting the preservation of either a particular cognitive content (i.e., trait self-knowledge) or a particular cognitive mechanism (i.e., a cognitive operation, allowing him to reach conclusions about his own traits despite the absence of trait self-knowledge). It may even be that accurate self-judgment, episodic and semantic memory impairments notwithstanding, recruits mechanisms that do not depend on declarative memory. Such mechanisms presumably only operate during self-evaluation, providing no help during the evaluation of other people.

## Introspective Computation

**Figure 1:**
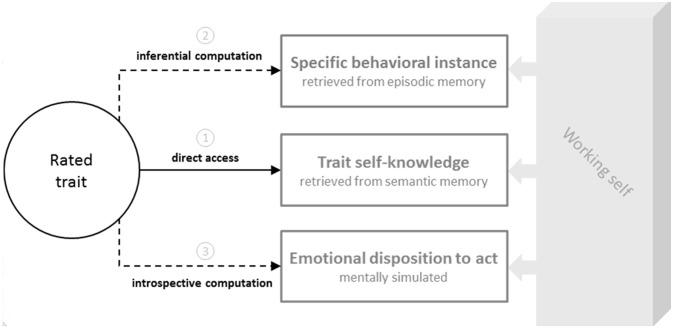
**Different strategies that can be used during self-evaluation.** When rating a given trait (e.g., *autonomous*), individuals can (1) directly compare it with trait self-knowledge stored in semantic memory (e.g., “I am an autonomous person” or “I am a dependent person”); (2) retrieve a specific behavioral instance linked to the rated trait from episodic memory (e.g., “The last time I encountered a problem with my video camera, I resolved it myself” or “When I bought my first video camera, I asked a friend to show me how to use it”) and use it to infer a general conclusion about themselves; or (3) mentally simulate their emotional disposition to act in a specific situation (e.g., “If I encountered a problem, I would first try to resolve it myself”), using it as an indicator of their general propensity to act in this way at all times, and thus of the trait they may or may not possess. The latter operation, which does not rely on personal information stored in either episodic or semantic declarative memory, is based on introspective computation.

Data reported in the literature suggest that self-evaluation essentially requires comparison of the rated trait with information retrieved from declarative memory. The latter may either already exist in the abstracted form of a trait (e.g., “I am an autonomous person”) stored in semantic memory, or else concern specific behavioral instances (e.g., “Last time I encountered a problem with my video camera, I resolved it myself”) stored in episodic memory that can be used to compute a general conclusion about oneself—an operation called *inferential computation*. However, on the strength of observations in amnesic syndromes, we would suggest that self-evaluation tasks requiring participants to judge the traits they currently possess can also be performed without retrieving personal information from declarative memory, be it episodic or semantic. Impairment of both of these memory systems is usually observed in patients with dementia, which also experience difficulties in accurately evaluating themselves. Although cases of patients producing accurate self-judgments despite episodic and semantic memory impairments are quite rare, they are not unheard of. We suggest that in these cases, when asked to rate a trait (e.g., *autonomous*) for self-descriptiveness, patients compare the trait to a mentally simulated content—an operation we call *introspective computation*. In other words, they imagine a situation that allows the trait to be verified (e.g., “How would I act if I encountered a problem for which I had no solution?”), and then mentally simulate their behavioral tendency in that situation. If this simulated tendency matches the trait being rated (e.g., “If I encountered a problem, I would start by trying to resolve it myself”), they conclude that they possess it (e.g., “I am indeed an autonomous person”). It is important to stress here that, from our point of view, of all the types of mental simulation the human brain is capable of (for a review see Decety and Grèzes, 2006), the mental simulation involved in introspective computation is quite specific. Unlike the episodic mental simulation of future scenarios conceptualized by Buckner and Carroll (2007) and Schacter et al. (2008), introspective computation cannot be described as episodic, as it essentially consists of a summary simulation (sustained by working memory) of our own affective reaction to particular circumstances. Instead of imagining a precise spatiotemporal context and a detailed unfolding of the situation, in this case we seek to capture our emotional reaction or spontaneous inclination to act in one way or another. In other words, we can use our current readiness to behave in a certain manner as an indicator of our general disposition to behave that way at all times, and thus conclude that we either do or do not possess a particular trait (see Figure 1).

Our mental simulation of how we would behave in an imagined situation and our everyday behavior presumably share the same foundation. We suggest that we possess an affective mental content regarding our relation to the world that is not necessarily conscious but, because it continuously governs our daily behavior, it may potentially be inferred from our actions and attitudes. This was conceptualized by Conway (2005) as the *working self*, that is, an active self-goal hierarchy regulating our affect, behavior and cognition, including the encoding, storage, retrieval and updating of information in declarative memory. This permanently active working self that strives to maintain our coherent and efficient functioning may also be called upon to guide our introspective computation, thereby allowing for the simulation of our behavioral tendencies as they would occur in real-life situations.

While introspective computation is one of the strategies that may be used to rate oneself on a trait, there remains the question of whether introspective computation alone can account for normal performances on self-evaluation tasks. This issue could be clarified if patients exhibiting a personality change were assessed shortly after that change, before episodic and semantic learning about their new personality had taken place. Their ability to rate themselves would then have to rely on introspective computation alone, as they would not have encoded enough information about themselves in declarative memory. To our knowledge, this kind of study has not so far been conducted. For now, researchers seem to agree only on the fact that episodic memories do not play a critical role in self-evaluation. By contrast, it is hard to say whether a semantic memory impairment spares accurate self-judgment because trait self-knowledge does not depend on it or because introspective computation allows for the mental simulation of one’s behavior in the absence of self-representations retrieved from episodic and semantic memory.

## Self-Evaluation in Patients Unaware of their Personality Changes

Amnesic patients who are aware of their cognitive deficits seem to accurately evaluate themselves, despite their episodic and sometimes even semantic memory impairments. Things become a lot more complicated when self-judgment is examined in patients who are unaware of their personality changes, as has been the case in several studies of patients with Alzheimer’s disease. In addition to episodic and semantic memory impairments, these patients exhibit a general cognitive decline that is accompanied by behavioral and psychiatric symptoms in the advanced stage, of which they seem to be unaware. While patients in the moderate and even advanced stages of Alzheimer’s disease are often able to complete self-evaluation tasks, providing the latter are adjusted to accommodate their cognitive deficits, they nonetheless do not seem to take their symptoms into account.

For instance, when Patient KR (Klein et al., 2003), who was unaware of her behavioral changes since being diagnosed with Alzheimer’s disease, was asked to evaluate herself on a list of traits, she provided a self-judgment that was inconsistent with her current behavior, as judged by her daughter and her caregiver at the assisted living facility, but consistent with her previous behavior, according to her daughter and son-in-law. Interestingly, KR seemed to possess preserved knowledge of her daughter’s traits, but not her caregiver’s, whom she had only known for the previous 2.5 years. These observations were interpreted by the authors of the study as reflecting a dysfunction of the patient’s semantic memory, namely her inability to encode the traits of recently met people as well as to update her trait self-knowledge. They suggested that this inability had not impacted the patient’s judgment of her daughter, whose behavior had not changed, but caused inaccurate self-evaluation, as the patient’s recent behavioral changes were not taken into account (Klein et al., 2003).

The hypothesis that inaccurate self-judgment in neurodegenerative diseases is due to a failure to update one’s personality traits was not, however, supported by recent findings in a group of patients in the initial stage of Alzheimer’s disease (Jedidi et al., 2014). In this study, the personality changes over the previous 10 years reported by patients were comparable to those reported by controls, indicating that the patients with Alzheimer’s disease perceived their long-term personality changes, without being aware of the recent and more dramatic ones induced by the disease. As the patients’ judgment of their current personality differed significantly from their relatives’ judgments of both their current personality and their past personality, they are unlikely to have based their current personality judgment on semantic knowledge about their previous personality (Jedidi et al., 2014). Furthermore, these findings are in line with data on amnesic syndromes, showing that episodic and semantic memory impairments alone do not impact patients’ accurate self-judgment, possibly because the latter can still rely on introspective computation. Why, therefore, was the self-judgment of KR, a patient with Alzheimer’s disease, not accurate?

We maintain that introspective computation is a very resistant mechanism, remaining operational despite declarative memory disorders. However, this cognitive operation only allows for accurate self-evaluation if: (1) patients’ behavior is stable, in which case their mentally simulated behavioral tendency is indeed illustrative of their general behavior on a daily basis; and (2) they can correctly judge their behavior, which requires them to be aware of the overall consequences of a given act and the way it would be judged by others. We therefore suggest two possible explanations for KR’s performance on the self-evaluation task, assuming that she based it essentially on introspective computation, owing to her memory impairments. First of all, it may be that her working self was undergoing changes resulting from her disease. Being unstable, it may have produced behavioral symptoms quite randomly and inconsistently. A temporary absence of the patient’s behavioral symptoms during the testing session may have led her to mentally simulate a healthy behavioral tendency, and thus to conclude that she did not have any behavioral or psychiatric symptoms. We favor a second explanation, however, whereby even if she was able to mentally simulate her behavioral tendency (i.e., as it would actually have occurred in a real-life situation), she may have had difficulty grasping its consequences, thus preventing her from negatively judging it, and from considering herself as possessing a negative trait in accordance with her negative behavior.

If individuals have difficulty grasping the implications of their deteriorating state or behavior, they will not see it as problematic and will therefore not integrate this negative connotation when judging themselves, even if their semantic memory is fully functioning. We therefore suggest that it was not KR’s memory difficulties *per se*, but her inability to accurately judge her behavior, that can best explain why her self-judgment continued to match her personality prior to the disease. This interpretation is supported by the finding that patients diagnosed with frontotemporal dementia, which does not involve any major memory impairment in the early stage, are usually completely unaware of their dramatic personality changes, whereas patients in the initial stage of Alzheimer’s disease, despite being far more affected by declarative memory decline, judge themselves far more accurately in terms of their traits (Rankin et al., 2005; Shany-Ur et al., 2014).

Data obtained in patients with cognitive and behavioral impairments show that in order to alter their self-judgments, which essentially means ending up with a more negative one, patients need to be aware of their deficits. For instance, one study showed that while patients in the early stage of Alzheimer’s disease remain aware of their symptoms, their self-evaluation on a list of traits tends to reveal a more negative self-view than that of controls (Addis and Tippett, 2004). By contrast, another group study showed that patients in the moderate or advanced stages of Alzheimer’s disease have a relatively positive perception of their traits and general behavior, compared with healthy controls and even feel more satisfied with themselves (Eustache et al., 2013). To sum up, the less patients with degenerative disease are aware of their symptoms, the less depressive they feel (for a review see Mograbi and Morris, 2014) and the more positively they perceive themselves (Naylor and Clare, 2008).

Nonetheless, episodic and semantic memory impairments do seem to affect patients’ self-perception, although their impact varies according to the task being used to assess it. Self-evaluation tasks can be completed even by patients with neurodegenerative diseases such as Alzheimer’s disease (Klein et al., 2003; Rankin et al., 2005; Ruby et al., 2009; Eustache et al., 2013; Martinelli et al., 2013; Jedidi et al., 2014; Shany-Ur et al., 2014), semantic dementia (Duval et al., 2012), or frontotemporal dementia (Rankin et al., 2005; Zamboni et al., 2010; Shany-Ur et al., 2014; Sollberger et al., 2014), and most of the time indicate that these patients possess a positive self-judgment comparable to that of healthy participants. When asked to freely describe themselves, however, patients with Alzheimer’s disease have been shown to produce fewer self-descriptive statements (Addis and Tippett, 2004; Eustache et al., 2013), which can partly be explained by additional impairment of cognitive functions such as self-initiated memory searching. However, these results may also indicate deterioration of declarative self-conception, for in the advanced stage of the disease, patients sometimes clearly formulate the difficulty they have bringing to mind a conscious and coherent representation of themselves, with some patients producing statements such as “I don’t remember myself”. On the basis of these observations, we suggest that, unlike self-description tasks, self-evaluation tasks can be completed using cognitive operations that are particularly resistant to amnesia, because they are not based on self-representations retrieved from declarative memory systems. While impaired judgment abilities may result in overly positive self-evaluation in patients with neurodegenerative diseases, the mechanism involved in self-evaluation *per se* (i.e., introspective computation) probably remains intact, explaining why patients can successfully perform this task, despite considerable difficulty saying who they are.

## Cerebral Underpinnings

The nature of the self has been widely discussed by researchers in different domains, and theoretical conceptions of the self remain a matter of debate. In this context, our understanding of the brain structures underlying the self is undermined by a lack of comprehensive knowledge of the cognitive operations the self involves. Nonetheless, new questions arising from the use of neuroimaging techniques have helped to shift the focus away from the self as content to the self as process (Northoff et al., 2011). Most neuroimaging studies now tackle the self by exploring two avenues: the neural operations mandatory for the emergence of the sense of self; and brain regions that are systematically associated with the processing of information about the self (for example see Abraham, 2013; D’Argembeau, 2013).

More recently, several studies have been conducted to measure brain activation patterns during self-evaluation tasks and see how they vary according to cultural or genetic factors. Data reported in the literature suggest that cultural differences regarding self-perception may modulate the level of activation in the medial prefrontal cortex (MPFC), temporoparietal junction, anterior cingulate cortex, precuneus and hippocampus during self-evaluation tasks (Zhu et al., 2007; Sul et al., 2012; Ma et al., 2014). Nonetheless, genes coding for neurotransmitters may influence these regions’ sensitivity to social factors. For instance, some genes coding for serotonin neurotransmission have been shown to prevent the modulation of neural activity during self-evaluation tasks in those brain regions that usually respond with different activation levels according to participants’ culturally determined self-perceptions (Ma et al., 2014). However, variations in brain activity according to participants’ self-perceptions may merely reflect differences in the cognitive operations used by participants during self-evaluation, such as first- or third-person perspective taking (Sul et al., 2012). These findings underline the need for a better understanding of the role played by neurotransmission systems and brain regions in different cognitive processes, as well as the actual cognitive processes that are involved in self-evaluation.

To our knowledge, introspective computation has never been directly assessed in patients with amnesic syndromes. However, functional imaging studies exploring self-evaluation in healthy volunteers could provide insights about the brain regions that support it. While information retrieved from declarative memory is used during other-evaluation as well as self-evaluation tasks, introspective computation, by definition, cannot apply to other people, and can only be used to evaluate the self. Therefore, the neural underpinnings of introspective computation could be captured by isolating brain activity specifically associated with self-judgment and absent during other-judgment on traits.

Several functional magnetic resonance imaging (fMRI) studies have shown that self-evaluation tasks elicit greater activity in the MPFC than other-evaluation tasks, whether the other person is a famous public figure (Kelley et al., 2002; Zhu et al., 2012; Dégeilh et al., 2015) or an individual who is personally known to the participant, such as a best friend (Heatherton et al., 2006) or mother (Zhu et al., 2007; Ray et al., 2010; but see also Schmitz et al., 2004; Ochsner et al., 2005; Vanderwal et al., 2008). The MPFC is known to be part of the default mode network (Raichle et al., 2001), encompassing brain regions that exhibit activity during the resting state. These brain regions seem to respond whenever a person is engaged in goal-directed tasks, by decreasing their activation relative to baseline (Gusnard et al., 2001). The greater activity in the MPFC during self-evaluation vs. other-evaluation tasks therefore means that this brain region exhibits a much weaker decrease in activity during self-evaluation tasks, compared with other-evaluation tasks.

While studies comparing brain activity during self- vs. other-evaluation tasks allow us to identify brain regions that are selectively activated during the processing of information about the self, they do not permit us to clearly state which cognitive operations are involved in these tasks. Therefore, findings regarding brain activation patterns may be interpreted in different ways when it comes to the cognitive operations they underpin, even if these interpretations remain a matter for speculation, in the absence of direct experimental evidence. First of all, authors seem to agree that these activations do not reflect semantic memory involvement during self-evaluation tasks, as semantic memory processes are characterized by a major decrease in activity in the MPFC (Kelley et al., 2002; Zysset et al., 2002). As episodic memory involvement results in a weaker decrease in MPFC activity than semantic memory involvement, Ray et al. (2010) suggested that self-evaluation tasks involve episodic memory retrieval to a greater extent than other-evaluation tasks. Nonetheless, this interpretation contradicts behavioral findings reported by studies measuring the reaction times of healthy volunteers, which show that episodic memories are accessed far more often during judgments of others than during self-judgments (Klein et al., 1992, 2001). While the cognitive operations sustained by the MPFC during self-evaluation tasks have yet to be elucidated, we suggest that another process besides the retrieval of personal information from episodic or semantic memory may be involved, namely introspective computation. The finding that activity in the MPFC is greater during the judgment of one’s own preferences (e.g., “I like Leipzig”) than during the retrieval of episodic memories (Zysset et al., 2002) suggests that this activity indicates participants’ reference to their immediate emotional state rather than the retrieval of episodic memories.

The MPFC has been hypothesized to sustain the processing of various kinds of self-referential information (Jenkins and Mitchell, 2011; D’Argembeau and Salmon, 2012), and its different areas to be linked to different cognitive functions (Murray et al., 2012). We suggest that introspective computation is at least partially sustained by the MPFC, but further research is needed to find out what links introspective computation to other cognitive tasks that recruit the MPFC. In order to improve its understanding, direct assessment of introspective computation in healthy controls and patients with memory disorders is needed, as well as a better theoretical conceptualization of the mental operations involved in each of the cognitive tasks contrasted in this type of functional neuroimaging studies.

## Conclusion

In this article, we introduced the concept of introspective computation as a possible explanation for why patients with semantic and/or episodic memory impairments are still able to accurately judge their personality traits. We suggest that introspective computation, which does not rely on personal information stored in semantic or episodic memory, is a cognitive strategy that consists in simulating one’s behavioral tendencies in a situation that exemplifies a given personality trait. Instead of simulating their behavior in a detailed manner, people who use introspective computation tend to refer to their current inclination or affective disposition to act in one way or another, and use it to determine whether or not they possess a particular personality trait. Experimental studies are needed to directly assess whether this kind of mental simulation is indeed preserved in patients with amnesia, and which brain regions support it.

## Author Contributions

AJ reviewed the literature and conceptualized the theoretical model aiming at explaining data obtained in studies in amnesia. PQ, ML, BD and FE contributed to the drafting, revision of the article and discussion of its important content, namely around the theoretical model proposed in the article. M-LE and FV contributed to the revision of the article and discussion of its important content, namely around the theoretical model proposed in the article.

## Funding

We would like to thank the Accident and Emergency Department, Neurology Units and Clinical Research Department of Caen University Hospital for their precious collaboration. This work was carried out as part of clinical research hospital programme undertaken by Caen University Hospital. The PhD funding of AJ was supported by Normandy Regional Council, the Vicq d’Azyr Association and INSERM.

## Conflict of Interest Statement

The authors declare that the research was conducted in the absence of any commercial or financial relationships that could be construed as a potential conflict of interest.
